# Efficacy of the spatial repellent product Mosquito Shield™ against wild pyrethroid-resistant *Anopheles arabiensis* in south-eastern Tanzania

**DOI:** 10.1186/s12936-023-04674-4

**Published:** 2023-08-30

**Authors:** Johnson Kyeba Swai, Alina Celest Soto, Watson Samuel Ntabaliba, Ummi Abdul Kibondo, Hassan Ahamad Ngonyani, Antony Pius Mseka, Anthony Ortiz, Madeleine Rose Chura, Thomas Michael Mascari, Sarah Jane Moore

**Affiliations:** 1 Vector Control Product Testing Unit, Environmental Health and Ecological Science Department, Ifakara Health Institute, Bagamoyo, Tanzania; 2 Department of Epidemiology and Public, Health Swiss Tropical and Public Health Institute, Allschwil, Switzerland; 3 University of Basel, Basel, Switzerland; 4 S. C. Johnson & Son, Inc., Racine, WI USA; 5 School of Life Sciences and Bio Engineering, The Nelson Mandela, African Institution of Science and Technology, Tengeru, Arusha, United Republic of Tanzania

**Keywords:** Spatial repellent, Emanator, *Anopheles*, Pyrethroid, Transfluthrin, Experimental hut

## Abstract

**Background:**

Spatial repellents that create airborne concentrations of an active ingredient (AI) within a space offer a scalable solution to further reduce transmission of malaria, by disrupting mosquito behaviours in ways that ultimately lead to reduced human-vector contact. Passive emanator spatial repellents can protect multiple people within the treated space and can last for multiple weeks without the need for daily user touchpoints, making them less intrusive interventions. They may be particularly advantageous in certain use cases where implementation of core tools may be constrained, such as in humanitarian emergencies and among mobile at-risk populations. The purpose of this study was to assess the efficacy of Mosquito Shield™ deployed in experimental huts against wild, free-flying, pyrethroid-resistant *Anopheles arabiensis* mosquitoes in Tanzania over 1 month.

**Methods:**

The efficacy of Mosquito Shield™ transfluthrin spatial repellent in reducing mosquito lands and blood-feeding was evaluated using 24 huts: sixteen huts were allocated to Human Landing Catch (HLC) collections and eight huts to estimating blood-feeding. In both experiments, half of the huts received no intervention (control) while the remaining received the intervention randomly allocated to huts and remained fixed for the study duration. Outcomes measured were mosquito landings, blood-fed, resting and dead mosquitoes. Data were analysed by multilevel mixed effects regression with appropriate dispersion and link function accounting for volunteer, hut and day.

**Results:**

Landing inhibition was estimated to be 70% (57–78%) [IRR 0.30 (95% CI 0.22–0.43); p < 0.0001] and blood-feeding inhibition was estimated to be 69% (56–79%) [IRR 0.31 (95% CI 0.21–0.44; p < 0.0001] There was no difference in the protective efficacy estimates of landing and blood-feeding inhibition [IRR 0.98 (95% CI 0.53–1.82; p = 0.958].

**Conclusions:**

This study demonstrated that Mosquito Shield™ was efficacious against a wild pyrethroid-resistant strain of *An. arabiensis* mosquitoes in Tanzania for up to 1 month and could be used as a complementary or stand-alone tool where gaps in protection offered by core malaria vector control tools exist. HLC is a suitable technique for estimating bite reductions conferred by spatial repellents especially where direct blood-feeding measurements are not practical or are ethically limited.

**Supplementary Information:**

The online version contains supplementary material available at 10.1186/s12936-023-04674-4.

## Background

Significant advancements against the global burden of malaria have been achieved in recent decades, with a 40% reduction in disease between year 2000 and 2015 [1]. Use of core vector control tools, specifically insecticide-treated nets (ITNs) and indoor residual spray (IRS), have brought about much of this change; an estimated 68% of cases averted are attributable to ITNs alone [1]. Recently however, progress against malaria cases and deaths has stalled in many countries, highlighting the urgent need to both strengthen implementation of existing core tools and bring to market new vector control tools that are safe, quality, and efficacious to fill gaps in protection and to continue incremental advancements toward malaria elimination [2].

Spatial repellents are one of a handful of vector control tools that hold promise as a scalable solution to further reduce transmission of malaria. Spatial repellents are products that create airborne concentrations of an active ingredient (AI) within a space, disrupting mosquito behaviours in ways that ultimately lead to a reduction in human-vector contact [3]. There is a large diversity of existing and emerging spatial repellent product forms globally. One of the most familiar and broadly available spatial repellent forms is the mosquito coil, which when ignited disperses an AI in the air through convection as the coil combusts. Liquid electric emanators and vaporizing mats also use heat to disperse an AI, but they require a source of electricity which may not be continuously available to populations most at risk of malaria [4]. Coils, liquid electrics, and vaporizing mats also rely on daily activation by end users.

Passive emanator spatial repellents rely solely on natural air movement to drive volatilization and dispersal of an AI from a dosed surface into a space without the need for heat or electricity [5]. When used indoors, passive emanator spatial repellents can protect multiple people within the treated space [6] and can last for multiple weeks without the need for daily user touchpoints, making them less intrusive interventions [7]. Passive spatial repellent products designed to be implemented by end users also may be particularly advantageous in certain use cases where implementation of core tools may be constrained, such as in humanitarian emergencies and among mobile at-risk populations [8].

Recently the impact of Mosquito Shield™ (a passive emanator spatial repellent) has been evaluated against malaria transmission in Indonesia, showing 27.7% protective efficacy (PE) against first-time malaria infections, (which was not statistically significant due in part to zero-to-low incidence in some clusters, undermining the ability to detect a protective effect), but a statistically significant 60% decrease in infection among a subset of 12 moderate- to high-risk clusters [9]. Mosquito Shield™ also was evaluated against dengue and Zika in Peru and showed a statistically significant 34.1% PE [10]. While the public health value of spatial repellents is being determined through previous and ongoing clinical trials [11, 12], it also is important to conduct robust evaluations of the entomological impact of spatial repellent products against disease vectors using methods that are appropriate and broadly implementable across disease-endemic countries.

The purpose of this study was to assess the efficacy of Mosquito Shield™ deployed in experimental huts against wild, free-flying, pyrethroid-resistant *Anopheles arabiensis* mosquitoes in Tanzania over 1 month.

## Methods

### Study location

This study was conducted between November and December 2019 at the Ifakara Health Institute (IHI) Ifakara Branch, Lupiro Field Station located in Lupiro village (8.385° S and 36.670° E) in Ulanga District, south-eastern Tanzania. The village lies 270 m above sea level on the Kilombero River valley, 26 km south of Ifakara town. Lupiro borders many small contiguous and perennially swampy rice fields to the northern and eastern sides. The annual rainfall is 1200–1800 mm with temperatures ranging between 20 and 33 °C. The principa malaria vectors in the area are pyrethroid-resistant *An. arabiensis*, and *Anopheles funestus *sensu stricto (s.s.) (*An. funestus* s.s. constitutes > 80% of the *An. funestus* complex) [13–15]. The mechanism of pyrethroid resistance is upregulation of mixed-function oxidases [14].

### Intervention

**Fig. 1:**
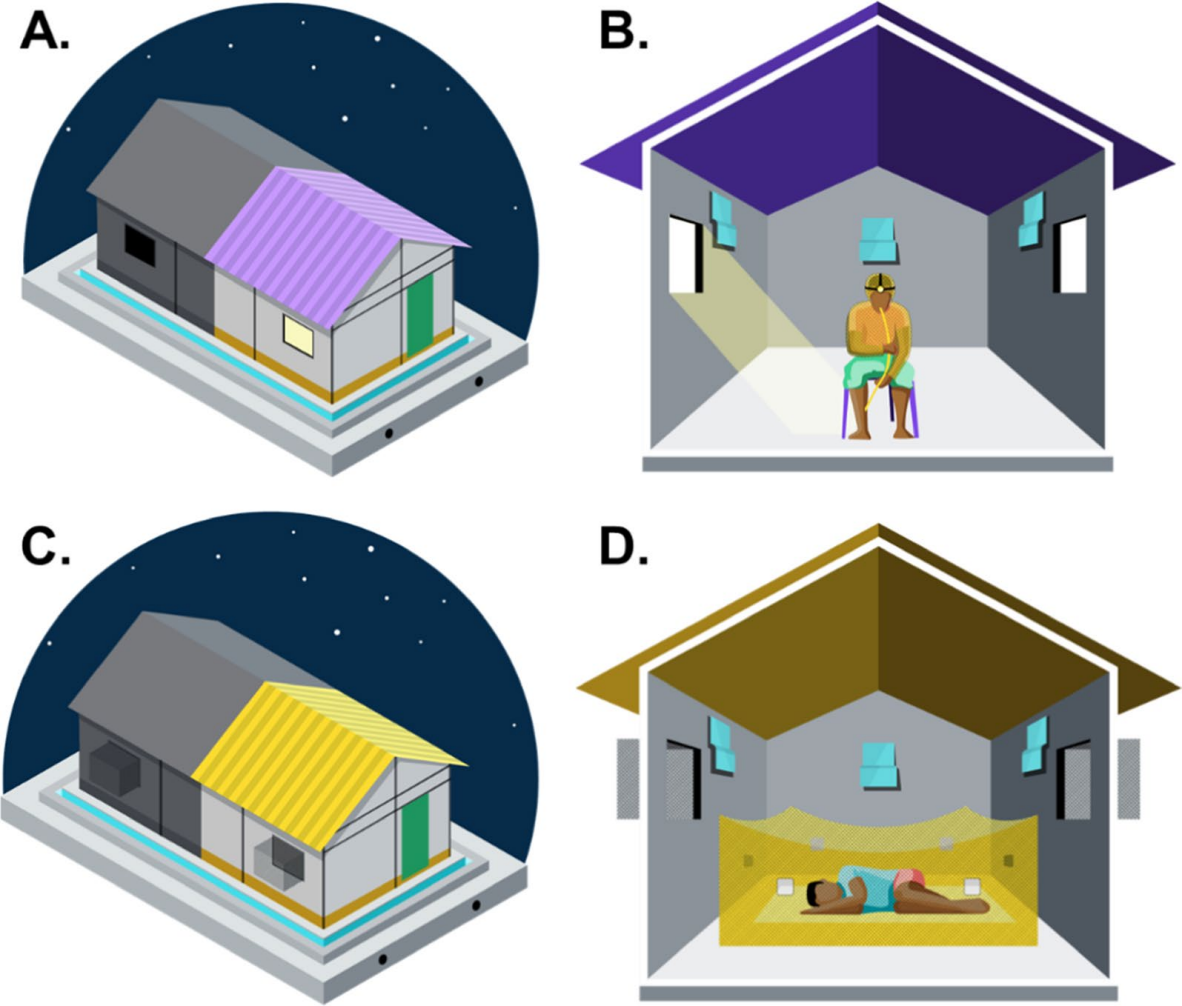
Set up of huts used for Human Landing Catch “Landing” (**A**, **B**) and classic experimental hut “Feeding” (**C**, **D**) experiments including the placement of the Mosquito Shield™ (**B**, **D**)

The Mosquito Shield™ spatial repellent is a folded 21.6 cm × 26.7 cm sheet of plastic film dosed with 110 mg of transfluthrin with a label claim of 30 days (SC Johnson & Son, Racine, WI, USA). Three Mosquito Shield™ products were placed according to manufacturer specifications at a height of 1.8 m from the ground and at centre length of each wall in each hut: one on each of the two walls with a window and one on the wall across from the door (Fig. 1). The Mosquito Shield™ products were installed at 16:00 h on the first day of the study and were removed after 32 days.

### Experimental huts

The study was conducted in “new” Ifakara experimental huts (NIEH), which are the same design as the original Ifakara experimental huts [16] but divided with a fully sealed plywood dividing wall to make two NIEH (Fig. 1). Hereafter in this manuscript NIEH will be referred to as “huts”. The dimensions of the huts are 3.25 m × 3.5 m × 2 m (length × width × height) with a gabled roof of 0.5 m apex and volume of 28.43 m^3^. Each hut had two windows and 10 cm-wide eave gaps. Baffles are fitted to the eave gaps so that mosquitoes can enter but not exit the huts. The two huts comprising each larger hut structure were assigned the same intervention to ensure there were no carry over effects of Mosquito Shield™ to untreated huts. Paired huts were approximately 20 m apart so that each block of two huts was independent.

### Study design

The efficacy of Mosquito Shield™ in reducing mosquito lands and blood-feeding was evaluated using a total of 24 huts: sixteen huts were allocated to Human Landing Catch (HLC) collections (*HLC experiment*) and eight huts to estimating the reduced blood-feeding (*Feeding experiment*). In both the HLC and Feeding experiments, half of the huts received no intervention (control) while the remaining received the intervention (three Mosquito Shield™ as described above). The treatments (Mosquito Shield™ or untreated control) were randomly allocated to huts using a random number generator and remained fixed in the huts for the full duration of the study.

### HLC experiment

Human landing catches were conducted for a total of 12 h (18:00 h to 06:00 h) every night for the duration of the experiment (32 nights) inside the huts, which had completely open windows (no exit traps) to allow mosquitoes to freely enter and exit (Fig. 1A, B). A total of 32 study participants were assigned to 16 pairs at the start of the experiment, and study participants remained in the same pair for the duration of the study. Each night, the 12-h collection period was divided into two 6-h shifts, with one study participant conducting HLC from 18:00 h to 00:00 h, and the other from 00:00 h to 06:00 h. The 16 pairs of study participants rotated to a new hut each night sequentially so that each pair of study participants collected mosquitoes in each hut twice over 32 consecutive nights. The two participants that moved as a pair did not swap between the two collection shifts. Those allocated to conduct HLC from 18:00 to 00:00 h did so from the beginning to end of the study. Once his round of data collection (18:00–00:00 h) finished, a tent was offered where he would sleep till morning (06:00 h) and then return home. This was also done for those whose shifts started from 00:00 to 06:00 h to ensure study participants are safe and do not have to travel at night to and fro the study site as well as maintain punctuality.

When conducting HLC, study participants wore shorts, closed shoes, and a net jacket covering the torso and head. Study participants sat on a chair placed in the centre of the hut and collected mosquitoes that landed on their bare lower legs for 50-min periods per hour by capturing them with an aspirator and transferring them into a netted paper cup. At the top of each hour participants took a break to maintain alertness as well as go to the bathroom if one needed. Captured mosquitoes were placed into different cups for each hour of collection. The following morning after 06:00 h, mosquitoes were transferred to the field laboratory, killed by freezing, then sorted and scored by species. Female mosquitoes were identified to species level using morphological keys [17] and a subsample of *Anopheles gambiae *sensu lato (s.l.) was sent for molecular identification [18, 19]. WHO tube tests using one times discriminating concentrations of pyrethroids were conducted on *An. arabiensis* collected from the field site at the time of the experiment according to WHO methods [20].

### Feeding experiment

The Feeding experiment also was conducted for a total of 12 h (18:00 h to 06:00 h) every night for 32 consecutive nights inside huts with exit traps on the windows to capture exiting mosquitoes (Fig. 1C, D). A total of eight study participants rotated sequentially through the huts each night so that each study participant slept in each hut four times over 32 consecutive nights. Study participants slept under an untreated bed net (SafiNet®, A to Z Textile Mills, Ltd., Arusha, Tanzania) deliberately holed with six 4 × 4 cm holes according to WHO ITN evaluation guidelines [21]. This was done to reduce biting pressure on study participants for their comfort and safety. Study participants entered the huts at 18:00 h every day and remained under the bed net until 06:00 h when they collected all live and dead mosquitoes from inside the bed net and window exit traps using a mouth aspirator, and from inside the hut using a Prokopack aspirator. Collected mosquitoes were transferred to the field laboratory for sorting and scoring by collection location (bed net, exit trap, or inside the hut) as dead unfed, dead fed, alive unfed or alive fed. Live mosquitoes were held at 27 ± 5 °C and provided access to 10% sucrose solution for up to 24 h to assess delayed mortality. Females were identified to species level using morphological keys [17].

### Statistical analysis

Data analysis was performed using STATA 16 software (StataCorp LLC, USA). Descriptive statistics were presented as William’s means [22] with 95% confidence intervals (95% CI). Analysis was performed only on *An. arabiensis* mosquitoes, hereafter mosquitoes.

The primary outcome measure of this study was protective efficacy (PE) estimated in terms of the reduction in the number of mosquitoes that landed on the study participants (landing inhibition) or the reduction in the number of blood-fed mosquitoes captured in the experimental hut (blood-feeding inhibition). The effect of Mosquito Shield™ on the number of mosquitoes landing or the number fed was examined using a multilevel mixed effects regression with a negative binomial distribution and log link. Intervention, volunteer and experimental night were fixed factors and hut was added as a random factor to account for clustering of observations. The PE for each experiment was calculated by (1 − IRR) * 100, where IRR is the incidence rate ratio in the Mosquito Shield™ group compared to the control.

The data was further analysed with intervention interacted with fixed factors for time (a variable representing blocks of 4 nights) to estimate efficacy as the intervention aged. Volunteer and experimental night were also included as fixed factors and hut was added as a random factor to account for clustering of observations.

The agreement between landing and blood-feeding PE estimates was explored using the same regression model with an interaction between intervention and technique (HLC/Feeding) with the number of landed or fed mosquitoes as the outcome. Volunteer and experimental night were also included as fixed factors and hut was added as a random factor to account for clustering of observations.

Additional outcomes indoor resting density, knockdown (KD), 24-h mortality (M24), induced exophily, and deterrence were estimated for Mosquito Shield™ from the Feeding experiment. KD was calculated as the proportion of mosquitoes that were collected dead in the morning. M24 was calculated as the proportion of dead mosquitoes after all mosquitoes (alive or KD) were held for 24-h. Daily KD and M24 were corrected by the control using Abbott’s Formula [23]. Reduction in indoor resting was calculated as the number of mosquitoes captured alive resting on walls in the morning in huts with Mosquito Shield™ relative to the untreated control. Induced exophily was calculated as the proportion of female mosquitoes captured in the exit traps out of the total number inside the hut and exit traps in the Mosquito Shield™ arm relative to the untreated control huts. Deterrence was calculated by comparing the total number of mosquitoes in huts in the Mosquito Shield™ arm relative to the controls. The effects of the intervention on these parameters were analysed using a multilevel mixed effects regression with a binomial error and logit link for proportional data (KD, M24, exophily), or a negative binomial error with a log link for count outcomes (deterrence, indoor resting) with volunteer and nights as fixed factors and hut as a random factor to account for clustering. The agreement between resting, landing and blood-feeding PE estimates was explored using the same regression model with an interaction between treatment arms and technique (HLC or Feeding compared to resting) with the number of landed or fed mosquitoes compared to live resting as the outcome.

## Results

Confirmatory sub-species identification using PCR was carried out on a total of 102 wild *An. gambiae* s.l. mosquitoes randomly selected throughout the study. The species complex comprised of 98.9% *An. arabiensis* (92/93 successful amplifications) and 1.1% *An. gambiae* s.s. (1/93 successful amplifications). Thus, the scoring of all *An. gambiae* s.l. mosquitoes collected during this study were scored as *An. arabiensis*. The numbers of *An. funestus* collected during the study were not sufficient to warrant their inclusion in the analysis. The *An. arabiensis* were found to be resistant to 1× discriminating concentrations of all pyrethroids tested and susceptible to all other insecticide classes (Additional file 1: Table S1).

### Landing inhibition

**Fig. 2:**
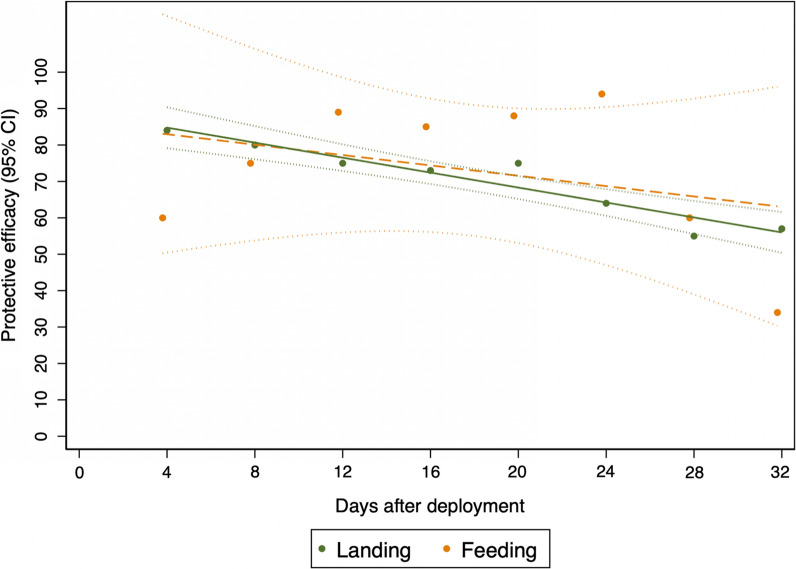
Trends in 4-days intervals of indoor landing and blood-feeding inhibition protective efficacy of the Mosquito Shield™ against wild pyrethroid-resistant *An. arabiensis*. Protective efficacy for each block of four days by landing (green) or feeding (orange). The lines show the model fitted reductions in landing (green) and fed mosquitoes (orange dashed) caught with 95% confidence intervals (dotted lines)

A total of 16,392 female *An. arabiensis* mosquitoes were captured by HLC in the control huts and 5591 in the Mosquito Shield™ huts over 32 nights, averaging 54 (50–58) and 15 (13–17) mosquitoes per hut per night, respectively. Landing inhibition was estimated to be 70% (57–78%), [IRR 0.30 (0.22, 0.43); p < 0.0001] Table 1. Landing inhibition reduced gradually as the intervention aged but remained above 50% at the end of the experiment (Fig. 2, Additional file 1: Table S2).

**Table 1:** Summary statistics and indoor protective efficacy of Mosquito Shield™ in reducing human landings and blood-feeding of wild pyrethroid-resistant *An. arabiensis*

Experiment	Intervention	^1^Total captured	^2^William’s Mean (95% CI)	^3^IRR (95% CI)	^4^PE (95% CI)	p-value
HLC	Control	16,392	53.8 (50.0, 57.8)	–	–	< 0.0001
	Mosquito Shield™	5591	14.8 (13.2, 16.7)	0.30 (0.22, 0.43)	70 (57, 78)	
Feeding	Control	195	2.0 (1.7, 2.2)	–	–	< 0.0001
	Mosquito Shield™	61	1.5 (1.2, 1.7)	0.31 (0.21, 0.44)	69 (56, 79)	
Resting	Control	48	0.24 (0.15, 0.33)	–	–	P = 0.001
	Mosquito Shield™	21	0.11 (0.06, 0.17)	0.39 (0.22, 0.68)	61 (32, 78)	

^1^Total captured refers total number of *An. arabiensis* that landed on participants during the HLC experiment and total number of blood-fed collected from the feeding experiment; ^2^Average caught per night per hut estimated as geometric mean due to skewness of mosquito count data; ^3^Incidence rate ratio (IRR) for intervention is reported from generalized negative binomial mixed effect model of mosquito landings/blood-fed adjusted for the effect of volunteer, hut location and study night. ^4^PE = Protective efficacy ((1-IRR) * 100); for landing inhibition is the percentage reduction in mosquito lands while for blood-feeding inhibition is the percentage reduction in number of blood-fed mosquitoes in the intervention relative to the control

### Blood-feeding inhibition

The total number of mosquitoes caught during the Feeding experiment was 1878 in the control and 1811 in the Mosquito Shield™ arm; of which, 195 blood-fed *An. arabiensis* mosquitoes were captured in the control huts and 61 in the Mosquito Shield™ huts. This corresponded to an overall 69% (95% CI 56–79%) blood-feeding inhibition, [IRR 0.31 (95% CI 0.21–0.44); p < 0.0001] (Table 1). Blood-feeding inhibition reduced gradually as the intervention aged but remained above 30% at the end of the experiment (Fig. 2, Additional file 1: Table S3). It should be noted that estimates of protective efficacy measured by feeding at 4-days intervals had wider confidence intervals than those of HLC due to low numbers of recaptured blood-fed mosquitoes.

### Comparison of the protective efficacy estimates between landing- and blood-feeding inhibition

Analysis of the interaction between the treatment arms and the method used (HLC or Feeding) tested using negative binomial regression, showed there was no difference in the estimates of landing and blood-feeding inhibition protective efficacy [IRR 0.98 (95% CI 0.53–1.82); p = 0.958].

### Indoor resting

Only 48 live indoor resting mosquitoes were collected in the negative control arm with 21 collected in the Mosquito Shield™ arm. This gave an estimated protective efficacy of 61% (95% CI 32–78%) that was significantly different [IRR 0.39 (95% CI 0.22–0.68); p < 0.001]. The PE measured by indoor resting was not significantly different to that measured by either landing [IRR 1.34 (95% CI 0.63–2.88); p = 0.448] or feeding [IRR 1.42 (95% CI 0.76–2.63); p = 0.268].

### Additional endpoints

Mosquito Shield™ also elicited knockdown: 40% (95% CI 35–45%) compared to 21% (95% CI 17–25%) in the control, corresponding to 28% (95% CI 25–32) control-corrected knockdown. After holding the mosquitoes for 24-h, significant mortality was also observed: 52% (95% CI 47–57%) compared to 35% (95% CI 31–40%) in the control. The control-corrected mortality at 24 h (i.e., mortality due to the intervention after factoring in control mosquito mortality) was 30% (95% CI 26–34%), which was significantly higher than in the control huts, OR 3.0 [(95% CI 2.3–3.9); p < 0.0001]. There was approximately 27% (95% CI 19–35%) increased exophily in the huts with the Mosquito Shield™ [OR 3.0 (95% CI 2.2–4.3); p < 0.0001] and 11% (95% CI 0–35%) overall reduced entry (deterrence) observed in the intervention huts as compared to the control huts, that was not statistically significant [IRR = 0.89 (95% CI 0.65,1.23); p = 0.495].

## Discussion

There is a need to generate robust evidence on the entomological impact of spatial repellents in different settings to generate data in support of product dossiers for review by the World Health Organization Vector Control Product Prequalification Team (WHO PQT) and country and regional regulatory authorities. This study assessed the entomological efficacy of Mosquito Shield™ against pyrethroid-resistant malaria vectors in south-eastern Tanzania over the length of life of the product.

Overall, the estimated values for the primary endpoints—landing inhibition and blood-feeding inhibition—support the efficacy of Mosquito Shield™ over 1 month. Mosquito Shield™ was found to reduce landings by *An. arabiensis* mosquitoes by > 70% (95% CI 57, 78). Similarly, the probability of a mosquito blood-feeding in huts was reduced by 69% (95% CI 56, 79%). The two methods of measuring personal protection can therefore be used interchangeably. To extend the methods that could be used for measuring the efficacy of spatial repellents under user conditions, such as in randomized controlled trials or in-home tests, indoor resting mosquitoes were also measured and found to measure a similar magnitude of effect (61%, 95% CI 32, 78) that was also not significantly different than the protective efficacy measured by either landing or feeding experiments in this highly controlled trial. However, the number of resting mosquitoes was extremely low which reduced the certainty of the estimate. A large study of Mosquito Shield™ in Peru conducted over 2 years also showed a significant reduction in indoor abundance of *Aedes aegypti* by 28.6% (one-sided 95% CI 24.1%) and blood-fed mosquitoes by 12.4% (one-sided 95% CI 4.2%) [10]. Other work in Tanzania has demonstrated reduction in *An. arabiensis* landings but not indoor densities in local homes using a 0.03% transfluthrin mosquito coil, although this was a very small trial over 24 weeks [24].

The findings from this study corroborate those from Belize [25] Indonesia [26, 27] Thailand [28, 29] Vietnam [30] as well as Tanzania [31–35] that demonstrated efficacy of transfluthrin-based spatial repellent products or prototypes against malaria vectors. The study further demonstrates that indoor use of a passive spatial repellent can induce several entomological responses that are known to impact malaria transmission by reducing the vectorial capacity of the mosquito [36]. Results of this study suggest significant impacts against many of these parameters, including blood-feeding inhibition, incapacitation (knock down and disarming) and mortality [37]. The mode of action of the intervention appeared to be at shorter range, interfering with host seeking rather than preventing mosquitoes from entering the huts. The ability of spatial repellents to elicit behaviours in mosquitoes that impact the vectorial capacity [38–40] and thus disease transmission [36] highlights that products such as Mosquito Shield™ could play a role in prevention of vector-borne diseases either as a complimentary tool [2, 41] to core interventions where gaps in protection may exist [8, 42] or as a stand-alone tool in situations where core vector control tools cannot be applied (for example in humanitarian emergency settings, or by mobile communities or forest workers who live in temporary structures) [8].

The rate of mosquito mortality appeared to increase throughout the experiment (data not shown). This suggests a potential build-up of transfluthrin concentration in the huts and its subsequent reduction as the intervention aged. However, as this trend was not observed in the Landing experiment, further work is ongoing to better understand how continuous re-deployment of Mosquito Shield™ differentially impacts efficacy over time.

In this study, two methods were used to measure the efficacy of Mosquito Shield™ against a wild population of *An. arabiensis:* human landing catches (HLC) and direct measurement of blood-feeding following methods used for ITN evaluation [21]. HLC is included in WHO guidelines on efficacy testing for both spatial repellents [43] and household insecticide products [44] as a standard method for evaluating product performance. HLC has the flexibility of being used in a variety of settings (including in-home tests during pilot implementations and operational research) as it does not require modifications to structures to enable capture of blood-fed mosquitoes. As structures are left in their normal-use state, there is no implicit impact on the emanation rates of the product or on the movement of mosquitoes due to experimental setup. As indoor resting used as a proxy for protective efficacy also aligned with estimates from HLC and feeding, it may be worthwhile to further evaluate this method as a means to test PE of spatial repellents under user conditions.

The design of the feeding experiment also enabled the direct measurement of blood feeding and induced mortality impact human-vector contact and vectorial capacity. The results of this study suggest that both HLC and the presented method adapted from Phase II ITN experimental hut tests are appropriate for evaluating the impact of indoor passive spatial repellents like Mosquito Shield™. Decisions by future researchers on which method to use could be made based on extrinsic factors, such as setting (e.g. in-home test or experimental hut), the endpoints of interest (e.g. personal protection or more comprehensive secondary/behavioural impacts that may also impact community protection), without compromise on validity of the results.

A potential limitation of this study is that the results are for one malaria vector in one location in Tanzania and therefore may not be representative of impact that may be seen in other transmission settings. Potential future work could include a comparison of the efficacy of Mosquito Shield™ from this study to results from other experimental hut types or in-home tests, and other geographies (with different vector species and levels of insecticide resistance). The control mortality in this study was also high, making the mortality estimates unreliable, which is why they were not further analysed. High control mortality was likely due to the mosquitoes being held in a room adjacent to a room where used Mosquito Shield™ were stored prior to disposal. It is advised to store volatile pyrethroids and other insecticides away from insectaries or holding rooms.

## Conclusion

This study demonstrated that Mosquito Shield™ was efficacious against a wild pyrethroid-resistant strain of *An. arabiensis* mosquitoes in Tanzania for up to 1 month. Data from this study also adds to the body of evidence that Mosquito Shield™ (and transfluthrin-based spatial repellents more broadly) could be used as a complementary or stand-alone tool where gaps in protection offered by core malaria vector control tools exist. Moreover, that vector control strategies utilizing volatile pyrethroids can be of public health importance in the fight against malaria and other disease transmitting mosquitoes given they elicit behaviours that directly impact vectorial capacity. HLC is a simple and suitable technique for estimating bite reductions conferred by spatial repellents especially where direct blood-feeding measurements are not practical or ethically limited. Indoor resting collections may also be used to estimate possible protective efficacy, although estimates were imprecise due to lower numbers of indoor resting mosquitoes in this scenario. Use of the standard ITN experimental hut study design in evaluating spatial repellents allows for a more precise estimation of blood-feeding inhibition as well as estimation of other entomological effects like mortality that substantially impact the vectorial capacity and ultimately disease transmission by mosquito vectors.

### Supplementary Information

**Additional file 1: Table S1.** Insecticide resistance profile of *Anopheles arabiensis *caught in experimental huts, south-eastern Tanzania. **Table S2.** Trend of protective efficacy (PE) of Mosquito Shield™ in reducing human landings of wild pyrethroid-resistant *An. arabiensis* over time. **Table S3.** Trend of protective efficacy (PE) of Mosquito Shield™ in reducing blood-fed wild pyrethroid-resistant *An. arabiensis* over time.

## Data Availability

The datasets used and or analysed in this study are available from the corresponding author upon reasonable request.

## Abbreviations

AI: Active ingredient
CI: Confidence interval
IRR: Incidence rate ratio
IRS: Indoor residual spraying
ITN: Insecticide-treated net
OR: Odds ratio
PE: Protective efficacy
SR: Spatial repellent
WHO: World Health Organization

## References

[1] Bhatt S, Weiss DJ, Cameron E, Bisanzio D, Mappin B, Dalrymple U (2015). The effect of malaria control on *Plasmodium falciparum* in Africa between 2000 and 2015. Nature.

[2] WHO (2022). Guidelines for malaria.

[3] Achee NL, Bangs MJ, Farlow R, Killeen GF, Lindsay S, Logan JG (2012). Spatial repellents: from discovery and development to evidence-based validation. Malar J.

[4] van Eijk AM, Ramanathapuram L, Sutton PL, Peddy N, Choubey S, Mohanty S (2016). The use of mosquito repellents at three sites in India with declining malaria transmission: surveys in the community and clinic. Parasit Vectors.

[5] Darbro JM, Muzari MO, Giblin A, Adamczyk RM, Ritchie SA, Devine GJ (2017). Reducing biting rates of *Aedes aegypti* with metofluthrin: investigations in time and space. Parasit Vectors.

[6] Tambwe MM, Saddler A, Kibondo UA, Mashauri R, Kreppel KS, Govella NJ (2021). Semi-field evaluation of the exposure-free mosquito electrocuting trap and BG-Sentinel trap as an alternative to the human landing catch for measuring the efficacy of transfluthrin emanators against *Aedes aegypti*. Parasit Vectors.

[7] Gryseels C, Uk S, Sluydts V, Durnez L, Phoeuk P, Suon S (2015). Factors influencing the use of topical repellents: implications for the effectiveness of malaria elimination strategies. Sci Rep.

[8] Wen S, Harvard KE, Gueye CS, Canavati SE, Chancellor A, Ahmed BN (2016). Targeting populations at higher risk for malaria: a survey of national malaria elimination programmes in the Asia Pacific. Malar J.

[9] Syafruddin D, Asih PBS, Rozi IE, Permana DH, Nur Hidayati AP, Syahrani L (2020). Efficacy of a spatial repellent for control of malaria in Indonesia: a cluster-randomized controlled trial. Am J Trop Med Hyg.

[10] Morrison AC, Reiner RC, Elson WH, Astete H, Guevara C, Del Aguila C (2022). Efficacy of a spatial repellent for control of Aedes-borne virus transmission: a cluster-randomized trial in Iquitos, Peru. Proc Natl Acad Sci USA.

[11] Ochomo EO, Gimnig JE, Bhattarai A, Samuels AM, Kariuki S, Okello G (2022). Evaluation of the protective efficacy of a spatial repellent to reduce malaria incidence in children in western Kenya compared to placebo: study protocol for a cluster-randomized double-blinded control trial (the AEGIS program). Trials.

[12] Van Hulle S, Sagara I, Mbodji M, Nana GI, Coulibaly M, Dicko A (2022). Evaluation of the protective efficacy of a spatial repellent to reduce malaria incidence in children in Mali compared to placebo: study protocol for a cluster-randomized double-blinded control trial (the AEGIS program). Trials.

[13] Kaindoa EW, Matowo NS, Ngowo HS, Mkandawile G, Mmbando A, Finda M (2017). Interventions that effectively target *Anopheles funestus* mosquitoes could significantly improve control of persistent malaria transmission in south-eastern Tanzania. PLoS ONE.

[14] Matowo NS, Munhenga G, Tanner M, Coetzee M, Feringa WF, Ngowo HS (2017). Fine-scale spatial and temporal heterogeneities in insecticide resistance profiles of the malaria vector, *Anopheles arabiensis* in rural south-eastern Tanzania. Wellcome Open Res.

[15] Lwetoijera DW, Harris C, Kiware SS, Dongus S, Devine GJ, McCall PJ (2014). Increasing role of *Anopheles funestus* and *Anopheles arabiensis* in malaria transmission in the Kilombero Valley, Tanzania. Malar J.

[16] Okumu FO, Moore J, Mbeyela E, Sherlock M, Sangusangu R, Ligamba G (2012). A modified experimental hut design for studying responses of disease-transmitting mosquitoes to indoor interventions: the ifakara experimental huts. PLoS ONE.

[17] Coetzee M (2020). Key to the females of Afrotropical *Anopheles* mosquitoes (Diptera: Culicidae). Malar J.

[18] Scott JA, Brogdon WG, Collins FH (1993). Identification of single specimens of the *Anopheles gambiae* complex by the polymerase chain reaction. Am J Trop Med Hyg.

[19] Koekemoer L, Kamau L, Hunt R, Coetzee M (2002). A cocktail polymerase chain reaction assay to identify members of the *Anopheles funestus* (Diptera: Culicidae) group. Am J Trop Med Hyg.

[20] WHO (2016). Test procedures for insecticide resistance monitoring in malaria vector mosquitoes.

[21] WHOPES (2013). Guidelines for laboratory and field testing of long-lasting insecticidal nets WHO/HTM/NTD/WHOPES/2013.3.

[22] Alexander N (2012). Review: analysis of parasite and other skewed counts. Trop Med Int Health.

[23] Abbott WS (1925). A method for computing the effectiveness of an insecticide. J Econom Entomol.

[24] Maia MF, Kreppel K, Mbeyela E, Roman D, Mayagaya V, Lobo NF (2016). A crossover study to evaluate the diversion of malaria vectors in a community with incomplete coverage of spatial repellents in the Kilombero Valley, Tanzania. Parasit Vectors.

[25] Wagman JM, Grieco JP, Bautista K, Polanco J, Briceño I, King R (2015). The field evaluation of a push-pull system to control malaria vectors in Northern Belize, Central America. Malar J.

[26] Syafruddin D, Bangs MJ, Sidik D, Elyazar I, Asih PB, Chan K (2014). Impact of a spatial repellent on malaria incidence in two villages in Sumba, Indonesia. Am J Trop Med Hyg.

[27] Syafruddin D, Asih PBS, Rozi IE, Permana DH, Nur Hidayati AP, Syahrani L (2020). Efficacy of a spatial repellent for control of malaria in Indonesia: a cluster-randomized controlled trial. Am J Trop Med Hyg.

[28] Sukkanon C, Bangs MJ, Nararak J, Hii J, Chareonviriyaphap T (2019). Discriminating lethal concentrations for transfluthrin, a volatile pyrethroid compound for mosquito control in Thailand. J Am Mosq Control Assoc.

[29] Sukkanon C, Nararak J, Bangs MJ, Hii J, Chareonviriyaphap T (2020). Behavioral responses to transfluthrin by *Aedes aegypti*, *Anopheles minimus*, *Anopheles harrisoni*, and *Anopheles dirus* (Diptera: Culicidae). PLoS ONE.

[30] Martin NJ, Nam VS, Lover AA, Phong TV, Tu TC, Mendenhall IH (2020). The impact of transfluthrin on the spatial repellency of the primary malaria mosquito vectors in Vietnam: *Anopheles dirus* and *Anopheles minimus*. Malar J.

[31] Mmbando AS, Ngowo H, Limwagu A, Kilalangongono M, Kifungo K, Okumu FO (2018). Eave ribbons treated with the spatial repellent, transfluthrin, can effectively protect against indoor-biting and outdoor-biting malaria mosquitoes. Malar J.

[32] Mwanga EP, Mmbando AS, Mrosso PC, Stica C, Mapua SA, Finda MF (2019). Eave ribbons treated with transfluthrin can protect both users and non-users against malaria vectors. Malar J.

[33] Ogoma SB, Ngonyani H, Simfukwe ET, Mseka A, Moore J, Killeen GF (2012). Spatial repellency of transfluthrin-treated hessian strips against laboratory-reared *Anopheles arabiensis* mosquitoes in a semi-field tunnel cage. Parasit Vectors.

[34] Masalu JP, Finda M, Okumu FO, Minja EG, Mmbando AS, Sikulu-Lord MT (2017). Efficacy and user acceptability of transfluthrin-treated sisal and hessian decorations for protecting against mosquito bites in outdoor bars. Parasit Vectors.

[35] Ogoma SB, Mmando AS, Swai JK, Horstmann S, Malone D, Killeen GF (2017). A low technology emanator treated with the volatile pyrethroid transfluthrin confers long term protection against outdoor biting vectors of lymphatic filariasis, arboviruses and malaria. PLoS Negl Trop Dis.

[36] Brady OJ, Godfray HC, Tatem AJ, Gething PW, Cohen JM, McKenzie FE (2016). Vectorial capacity and vector control: reconsidering sensitivity to parameters for malaria elimination. Trans R Soc Trop Med Hyg.

[37] Denz A, Njoroge MM, Tambwe MM, Champagne C, Okumu F, van Loon JJA (2021). Predicting the impact of outdoor vector control interventions on malaria transmission intensity from semi-field studies. Parasit Vectors.

[38] Bibbs CS, Hahn DA, Kaufman PE, Xue RD (2018). Sublethal effects of a vapour-active pyrethroid, transfluthrin, on *Aedes aegypti* and *Ae. albopictus* (Diptera: Culicidae) fecundity and oviposition behaviour. Parasit Vectors.

[39] Buhagiar TS, Devine GJ, Ritchie SA (2017). Effects of sublethal exposure to metofluthrin on the fitness of *Aedes aegypti* in a domestic setting in Cairns, Queensland. Parasit Vectors.

[40] Ogoma SB, Lorenz LM, Ngonyani H, Sangusangu R, Kitumbukile M, Kilalangongono M (2014). An experimental hut study to quantify the effect of DDT and airborne pyrethroids on entomological parameters of malaria transmission. Malar J.

[41] Bibbs CS, Kaufman PE (2017). Volatile pyrethroids as a potential mosquito abatement tool: a review of pyrethroid-containing spatial repellents. J Integr Pest Manag.

[42] Monroe A, Moore S, Koenker H, Lynch M, Ricotta E (2019). Measuring and characterizing night time human behaviour as it relates to residual malaria transmission in sub-Saharan Africa: a review of the published literature. Malar J.

[43] WHOPES (2013). Guidelines for efficacy testing of spatial repellents.

[44] WHO (2009). Guidelines for efficacy testing of household insecticide products: mosquito coils, vaporizer mats, liquid vaporizers, ambient emanators and aerosols.

