# Supplementary material for: Efficacy of the spatial repellent product Mosquito Shield™ against wild pyrethroid-resistant Anopheles arabiensis in south-eastern Tanzania
Source: Malar J. 2023 Aug 30;22:249. doi: 10.1186/s12936-023-04674-4 (PMC10466708; doi:10.1186/s12936-023-04674-4)
Supplement: Supplementary file 1 — Additional file 1: Table S1. Insecticide resistance profile of Anopheles arabiensis caught in experimental huts, south-eastern Tanzania. Table S2. Trend of protective efficacy (PE) of Mosquito Shield™ in reducing human landings of wild pyrethroid-resistant An. arabiensis over time. Table S3. Trend of protective efficacy (PE) of Mosquito Shield™ in reducing blood-fed wild pyrethroid-resistant An. arabiensis over time. [file 12936_2023_4674_MOESM1_ESM.docx]

**Additional file 1**

**Table S1** Insecticide resistance profile of *Anopheles arabiensis* caught in experimental huts, south-eastern Tanzania

| **Insecticide** | **% Mean 24-hour Mortality** |
| --- | --- |
| 0.05% Lambda cyhalothrin | 57 |
| 0.75% Permethrin | 72 |
| 0.05% Deltamethrin | 71 |
| 0.05% Alphacypermethrin | 37 |
| 4% DDT | 97 |
| 0.1% Bendiocarb | 100 |
| 0.25% Pirimiphos methyl | 100 |

**Table S2** Trend of protective efficacy (PR) of Mosquito Shield™ in reducing human landings of wild pyrethroid-resistant *An. arabiensis* over time.

| **Days post-placement** | **Intervention** | **Total captured** | **^1^ Williams**  **Mean (95% CI)** | **^2^IRR (95% CI)** | **^3^PE (95% CI)** | **p-value** |
| --- | --- | --- | --- | --- | --- | --- |
| 0-4 | Control | 1126 | 30 (22,39) | - | - | <0.0001 |
|  | Mosquito Shield™ | 199 | 4 (3,7) | 0.16 (0.12, 0.22) | 84 (78, 88) |  |
| 5-8 | Control | 2143 | 61 (50,76) | - | - | <0.0001 |
|  | Mosquito Shield™ | 459 | 11(7,17) | 0.20 (0.13, 0.32) | 80 (68, 87) |  |
| 9-12 | Control | 1766 | 49(39,62) | - | - | <0.0001 |
|  | Mosquito Shield™ | 453 | 12(9,16) | 0.25 (0.18, 0.36) | 75 (64, 82) |  |
| 13-16 | Control | 1350 | 40(33,47) | - | - | <0.0001 |
|  | Mosquito Shield™ | 369 | 10(8,13) | 0.27 (0.19, 0.37) | 73 (63, 81) |  |
| 17-20 | Control | 1566 | 43(34,55) | - | - | <0.0001 |
|  | Mosquito Shield™ | 500 | 12(8,18) | 0.25 (0.15, 0.40) | 75 (60, 85) |  |
| 21-24 | Control | 3035 | 83(64,108) | - | - | <0.0001 |
|  | Mosquito Shield™ | 1028 | 26(18,37) | 0.36 (0.25, 0.52) | 64 (48,75) |  |
| 25-28 | Control | 2586 | 71(55,91) | - | - | <0.0001 |
|  | Mosquito Shield™ | 1235 | 32(23,44) | 0.45 (0.31, 0.64) | 55 (36, 69) |  |
| 29-32 | Control | 2820 | 80(65,99) | - | - | <0.0001 |
|  | Mosquito Shield™ | 1348 | 38(30,48) | 0.43 (0.33, 0.57) | 57 (43, 67) |  |

Average caught per night per hut estimated as ^1^William’s mean due to skewness of mosquito count data; ^2^Incidence rate ratio (IRR) for intervention is estimated from generalized negative binomial mixed effect model of number of mosquitoes landing on a participant adjusted for the effect of volunteer, hut location and study night. ^3^PE = Protective efficacy ((1-IRR) *100) is the percentage reduction in number of mosquitoes landing on a participant in the intervention relative to the control estimated from the regression model.

**Table S3** Trend of protective efficacy (PR) of Mosquito Shield™ in reducing blood-fed wild pyrethroid-resistant *An. arabiensis* over time.

| **Days post-placement** | **Intervention** | **Total captured** | **^1^ Williams**  **Mean (95% CI)** | **^2^IRR (95% CI)** | **^3^PE (95% CI)** | **p-value** |
| --- | --- | --- | --- | --- | --- | --- |
| 0-4 | Control | 10 | 0.5 (0.2, 0.8) |  |  | 0.121 |
|  | Mosquito Shield™ | 4 | 0.2 (0, 0.4) | 0.40 (0.13, 1.28) | 60 (0, 87) |  |
| 5-8 | Control | 20 | 1 (0.5, 1.6) |  |  | 0.013 |
|  | Mosquito Shield™ | 3 | 0.1 (0, 0.3) | 0.25 (0.08, 0.75) | 75 (25, 92) |  |
| 9-12 | Control | 17 | 0.8 (0.3, 1.3) |  |  | 0.003 |
|  | Mosquito Shield™ | 2 | 0.1 (0, 0.2) | 0.11 (0.03, 0.48) | 89 (52, 97) |  |
| 13-16 | Control | 32 | 1.3 (0.6, 2.3) |  |  | 0.002 |
|  | Mosquito Shield™ | 2 | 0.1 (0, 0.2) | 0.15 (0.04, 0.50) | 85 (50, 96) |  |
| 17-20 | Control | 40 | 1.9 (1.1, 3.0) |  |  | 0.004 |
|  | Mosquito Shield™ | 16 | 0.7 (0.3, 1.2) | 0.12 (0.03, 0.51) | 88 (49, 97) |  |
| 21-24 | Control | 42 | 2.0 (1.1, 3.2) |  |  | 0.001 |
|  | Mosquito Shield™ | 28 | 1.3 (0.7, 2.2) | 0.06 (0.01, 0.29) | 94 (71, 99) |  |
| 25-28 | Control | 16 | 0.8 (0.4, 1.3) |  |  | 0.006 |
|  | Mosquito Shield™ | 4 | 0.2 (0.0, 0.4) | 0.40 (0.21, 0.77) | 60 (23, 79) |  |
| 29-32 | Control | 18 | 0.9 (0.4, 1.4) |  |  | 0.109 |
|  | Mosquito Shield™ | 2 | 0.1 (0.0, 0.2) | 0.66 (0.40, 1.10) | 34 (0, 60) |  |

Average caught per night per hut estimated as ^1^William’s mean due to skewness of mosquito count data; ^2^Incidence rate ratio (IRR) for intervention is estimated from generalized negative binomial mixed effect model of number of mosquitoes landing on a participant adjusted for the effect of volunteer, hut location and study night. ^3^PE = Protective efficacy ((1-IRR) *100) is the percentage reduction in number of mosquitoes landing on a participant in the intervention relative to the control estimated from the regression model
